# An R package for generic modular response analysis and its application to estrogen and retinoic acid receptor crosstalk

**DOI:** 10.1038/s41598-021-86544-0

**Published:** 2021-03-31

**Authors:** Gabriel Jimenez-Dominguez, Patrice Ravel, Stéphan Jalaguier, Vincent Cavaillès, Jacques Colinge

**Affiliations:** 1 Inserm U1194, Institut de Recherche en Cancérologie de Montpellier, Montpellier, France; 2 University of Montpellier, Montpellier, France; 3 ICM, Institut régional du Cancer de Montpellier, 208 avenue des Apothicaires, 34298 Montpellier cedex 5, France

**Keywords:** Computational biology and bioinformatics, Computational models, Gene regulatory networks, Genome informatics

## Abstract

Modular response analysis (MRA) is a widely used inference technique developed to uncover directions and strengths of connections in molecular networks under a steady-state condition by means of perturbation experiments. We devised several extensions of this methodology to search genomic data for new associations with a biological network inferred by MRA, to improve the predictive accuracy of MRA-inferred networks, and to estimate confidence intervals of MRA parameters from datasets with low numbers of replicates. The classical MRA computations and their extensions were implemented in a freely available R package called aiMeRA (https://github.com/bioinfo-ircm/aiMeRA/). We illustrated the application of our package by assessing the crosstalk between estrogen and retinoic acid receptors, two nuclear receptors implicated in several hormone-driven cancers, such as breast cancer. Based on new data generated for this study, our analysis revealed potential cross-inhibition mediated by the shared corepressors NRIP1 and LCoR. We designed aiMeRA for non-specialists and to allow biologists to perform their own analyses.

## Introduction

Modular response analysis (MRA) was introduced to infer the directions and strengths of connections between components of a biological system in a steady-state^1^. This approach can be applied to components at different levels of detail, *e.g.*, individual genes or subsystems, such as pathways or processes. MRA is based on the perturbation of individual components, the so-called modules. Various developments of MRA and related methods were recently reviewed^2^. In this report, we applied and extended MRA methodology to explore the crosstalk between two nuclear receptors (NRs). This resulted in the implementation of a new R^3^ package called “aiMeRA”.

Estrogen receptors (ERs) belong to the NR superfamily, which function as transcription factors, and are activated upon ligand binding. Both ER isoforms (ERα and ERβ) are involved in the control of cell proliferation and exhibit essential functions in tissue development and homeostasis, particularly in organs related to reproduction^4^. ERα overexpression is frequently observed in breast, ovarian, endometrial, and other hormone-driven tumors. The transcriptional activity of ERs is modulated by several coregulatory complexes, including coactivators and corepressors^4^. In the presence of estrogens or any agonist ligand, ERs interact preferentially with coactivators, or with a specific subclass of corepressors, including the nuclear receptor-interacting protein 1 (NRIP1, often named RIP140) and the ligand-dependent corepressor (LCoR). NRIP1 is a corepressor of particular interest because its expression is directly induced by estrogen, *i.e.*, NRIP1 installs a negative feedback loop to maintain ER signaling under control^5^. Indeed, abnormal NRIP1 expression is observed in ER-driven tumors^6,7^. LCoR represses the transcription of estrogen-induced gene expression^8^, and NRIP1 expression has been shown necessary for LCoR inhibitory activity in breast cancer (BC) cells^9^.

Interestingly, NRIP1 and LCoR function as corepressors for several ligand-dependent NRs. For instance, LCoR can repress the vitamin D receptor (VDR), retinoic acid receptors (RARs), but also RXR ligand-dependent transcription^8^ in addition to ERs. Moreover, NRIP1 is a known direct target and negative regulator of RAR transcription^10^.

There is experimental evidence of crosstalk between ER and RAR signaling^11^. For instance, ERα can suppress the basal expression of retinoic acid (RA)-responsive gene RARβ2, but also turns out to be necessary for its RA induction^12^. It was also found that ERα activates RARα1 expression in BC cells^13^. Other authors intersected RAR targets identified by chromatin immunoprecipitation-sequencing (ChIP-seq) with ER binding sites and discovered a significant overlap^14^. This study suggested a mechanism of space competition for ER and RA crosstalk in BC cells. A potential cooperative interaction between RARα and ER was also shown in BC cells^15^. Since NRIP1 and LCoR expression can both be regulated by RAR and ER transcription, we can further hypothesize that these molecules mediate part of ER-RAR crosstalk. The induced expression of NRIP1 and LCoR by one receptor produces molecules able to subsequently repress signaling of both receptors.

The first objective of this study was hence to explore the ER-RAR crosstalk at the transcriptional level by characterizing the ER-RAR-NRIP1-LCoR network by means of transcript abundance measurements and MRA. We considered a steady-state condition in a BC (MCF7-derived) cell line that would model BC cells under hormonal stimulation. We conducted perturbation experiments to generate quantitative PCR (qPCR) and mRNA sequencing (RNA-seq) data.

Given the nature of ER and RAR, *i.e.*, transcription factors, and the general ability of MRA to perform predictions^16^, we introduced two extensions of MRA in a second part of this study. We exploited the RNA-seq data and tested whether the ER-NRIP1-LCoR network inferred by MRA could be used to search for novel genes under strong ER transcriptional control. Then, we used the same ER-NRIP1-LCoR network to predict the expression levels of E2-regulated and ER target genes that were not perturbed during the construction of the MRA-inferred network. These variations from classical MRA inference, but also a new procedure to estimate confidence intervals (CIs) around MRA parameter estimates from a very low number of replicates, are available in the R package developed in this study.

Based on the application of multivariate calculus, we also present a straightforward mathematical derivation of MRA.

To put our work in perspective, other authors have extended the classical MRA approach, *e.g.*, by introducing Bayesian variable selection to better infer pathway topologies^17^, by pruning edges for a similar purpose^16^, or by dealing with incomplete perturbation sets^18–20^. The Blüthgen Laboratory recently released another R package to perform MRA computations^21^ with a specific focus on their edge-pruning and associated maximum likelihood extension of MRA^16^.

## Methods

### Mathematical derivation

**Figure 1:**
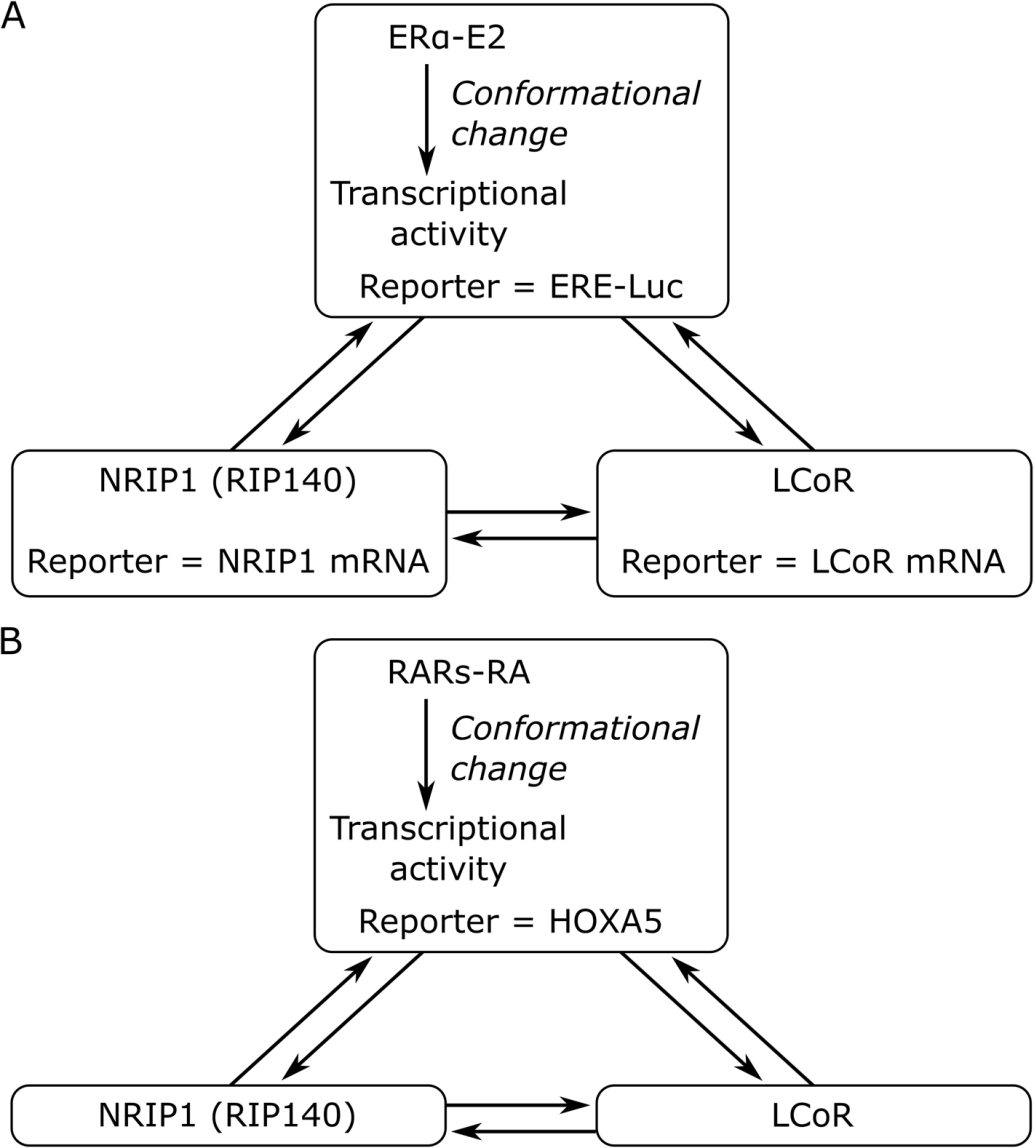
MRA general principle. (**A**) The ERα-NRIP1-LCoR transcriptional network. The activity level of each module is given by a measured reporter. Connection coefficients (edge weights) are determined from perturbation experiments. (**B**) The RARs-NRIP1-LCoR transcriptional network. Figure created with Inkscape 0.92 (www.inkscape.org).

The seminal MRA publication by Kholodenko and colleagues introduced the concept of inferring interdependencies (connection coefficients or local response coefficients) modularly within a biological system^1^. That is, subsystems comprised of molecules and their relationship at a detailed level, which is outside the scope of this study, could be captured as a single module with one measurable quantity describing the overall module activity. For instance, in the case of ERα signaling, the complex process of ligand binding and transcriptional activity can be represented by a single module (Fig. 1A); the activity of this module is measured in MELN cells by the reporter gene luciferase. NRIP1 and LCoR activities were determined by their respective mRNA abundances. In this study, we assessed the dependence of each module activity with respect to that of the others; we computed connection coefficients to assign the directed edges of Fig. 1A. The solution was found in a steady-state after applying successive elementary perturbations on each module activity. Depending on the application, this framework can be applied to different molecular species and processes, *e.g.*, protein/metabolite concentrations or protein phosphorylation levels^2^.

Now, in full generality, we assume that there are $n$ modules whose activities are given by $x\in {\mathbb{R}}^{n}$. We further admit the existence of $n$ intrinsic parameters, $p\in {\mathbb{R}}^{n}$, one per module, each of them perturbed by elementary perturbations. One can imagine mRNA abundance parameters for perturbations, such as siNRIP1 or siLCoR, and the number of available ERα-E2 bound complexes for the E2 perturbation. In other circumstances, perturbations may change affinity constants or other physical parameters. Lastly, we assume that there exists $S{\subset {\mathbb{R}}}^{n}\times {\mathbb{R}}^{n}$, an open subset, and $f:S  \to  {\mathbb{R}}^{n}$ of class ${\mathcal{C}}^{1}$, *i.e.*, continuously differentiable, such that.

1 $$ \dot{x} = f\left( {x,p} \right). $$

We do not need to know $f\left( {x,p} \right) = \left( {f_{1} \left( {x,p} \right), \ldots ,f_{n} \left( {x,p} \right)} \right)^{t}$ explicitly, but we need one more hypothesis that is the existence of a time $T>0$ such that all the solutions we consider for any $p$ and initial conditions of $x$, have reached a steady-state, *i.e.*,

$$\dot{x}=0, \forall \mathrm{t}>\mathrm{T}.$$

The unperturbed, basal state of the modules is denoted $x({p}^{0}){\in {\mathbb{R}}}^{n}$ and it has corresponding parameters ${p}^{0}{\in {\mathbb{R}}}^{n}$. According to our hypotheses, $f\left(x({p}^{0}),{p}^{0}\right)=0\Leftrightarrow {f}_{i}\left({x(p}^{0}),{p}^{0}\right)=0, \forall i\in$. By the implicit function theorem, $\forall i$, there exists open neighborhoods $V_{i} \times W_{i} \subset {\mathbb{R}}^{n - 1} \times  \times {\mathbb{R}}^{n}$ of $\left( {x_{1}^{0} , \ldots ,x_{i - 1}^{0} ,x_{i + 1}^{0} , \ldots ,x_{n}^{0} ,p_{1}^{0} , \ldots ,p_{n}^{0} } \right)$ and ${U}_{i}\subset {\mathbb{R}}$ of ${x}_{i}^{0}$, as well as ${g}_{i}:{V}_{i}\times {W}_{i}\to {U}_{i}$ (also ${\mathcal{C}}^{1}$) with

2 $$ f_{i} \left( {x_{1} , \ldots ,x_{i - 1} ,g_{i} \left( \cdots \right),x_{i + 1} , \ldots ,x_{n} ,p_{1} , \ldots ,p_{n} } \right) = 0,\quad \forall \left( {x_{1} , \ldots ,x_{i - 1} ,x_{i + 1} , \ldots ,x_{n} ,p_{1} , \ldots ,p_{n} } \right) \in V_{i} \times W_{i} $$

We denote $x({p}^{0}+\Delta p)$, the steady-state corresponding to the changed parameters ${p}^{0}+\Delta p$, *i.e.*, the solution of $\dot{x}\left({p}^{0}+\Delta p\right)=f(x\left({p}^{0}+\Delta p\right),{p}^{0}+\Delta {p}^{0})$. We introduce the notation ${x}_{j\ne i}$ to denote all the ${x}_{j}$ but ${x}_{i}$. Now, if we assume that $({x}_{j\ne i}\left({p}^{0}+\Delta p\right),{p}^{0}+\Delta p)$ belong to ${V}_{i}\times {W}_{i}$ for all the perturbations considered experimentally, then by Taylor’s Formula3

Dividing each side by ${x}_{i}\left({p}^{0}\right),$ Eq. (3) can be rewritten4

Since the parameter ${p}_{j}$ influences the module $j$ only, $\frac{\partial {g}_{i}}{\partial {p}_{j}}=0$ if $j\ne i$. Moreover, ${g}_{i}({x}_{j\ne i},p) ={x}_{i}({x}_{j\ne i},p)$ in ${V}_{i}\times {W}_{i}$, and if we denote

$$\frac{\Delta {x}_{i}}{{\mathrm{x}}_{\mathrm{i}}}=\frac{{x}_{i}\left({p}^{0}+\Delta p\right)-{x}_{i}\left({p}^{0}\right)}{{x}_{i}\left({p}^{0}\right)},$$

and

5 $$ r_{i,j} = \frac{{x_{j} \left( {p^{0} } \right)}}{{x_{i} \left( {p^{0} } \right)}}\frac{{\partial x_{i} }}{{\partial x_{j} }}\left( {p^{0} } \right),\quad j \ne i, $$

Then6

We next consider elementary perturbations $q_{k}$, $k \in \left\{ {1, \ldots ,n} \right\}$, which only influence the module $k$, *i.e.*, the parameter ${p}_{k}$. Neglecting the second-order term  and writing

$${\left(\frac{\Delta {x}_{i}}{{x}_{i}}\right)}_{{q}_{k}}$$

the relative difference in activity of module $i$ upon $\Delta {p}_{k}$ change induced by perturbation ${q}_{k}$, we find

7 $$ \left( {\frac{{{\Delta }x_{i} }}{{x_{i} }}} \right)_{{q_{k} }} = \mathop \sum \limits_{j \ne i} r_{i,j} \left( {\frac{{{\Delta }x_{j} }}{{x_{j} }}} \right)_{{q_{k} }} ,\quad k \ne i, $$

8 $$ \left( {\frac{{{\Delta }x_{i} }}{{x_{i} }}} \right)_{{q_{i} }} = \mathop \sum \limits_{j \ne i} r_{i,j} \left( {\frac{{{\Delta }x_{j} }}{{x_{j} }}} \right)_{{q_{k} }} + \frac{{\partial x_{i} }}{{\partial p_{i} }}\left( {p^{0} } \right)\left( {\frac{{{\Delta }p_{i} }}{{x_{i} }}} \right). $$

By defining ${r}_{i,i}=-1$, we can write Eqs. (7) and (8) in matrix form:

9 $$ rR = - P, $$

where $R$ is the matrix of systems-level (relative) changes of modules to perturbations $R_{j,k} = \left( {\frac{{{\Delta }x_{j} }}{{x_{j} }}} \right)_{{q_{k} }} ,\quad j,k \in \left\{ {1, \ldots ,n} \right\}$. $P$ is a diagonal matrix with $P_{i,i} = \frac{{\partial x_{i} }}{{\partial p_{i} }}\left( {p^{0} } \right)\left( {\frac{{{\Delta }p_{i} }}{{x_{i} }}} \right)$, $i \in \left\{ {1, \cdots ,n} \right\}$. The system (9) can be solved in two steps^1^. Firstly, $r=-P{R}^{-1}$ and since ${r}_{i,i}=-1$, we have ${P}_{i,i}{\left({R}^{-1}\right)}_{i,i}=1$, thus ${P}_{i,i}=\frac{1}{{\left({R}^{-1}\right)}_{i,i}}$. Secondly,

$$ r = - \left[ {{\text{diag}}\left( {R^{ - 1} } \right)} \right]^{ - 1} R^{ - 1} . $$

The elements of $R$ are defined as ${\left(\frac{{x}_{i}\left({p}^{0}+\Delta {p}_{k}\right)-{x}_{i}\left({p}^{0}\right)}{{x}_{i}\left({p}^{0}\right)}\right)}_{{q}_{k}}$, but as suggested previously^1^, we rather estimated this quantity by

10 $$ R_{j,k} = 2\left( {\frac{{x_{i} \left( {p^{0} + {\Delta }p_{k} } \right) - x_{i} \left( {p^{0} } \right)}}{{x_{i} \left( {p^{0} + {\Delta }p_{k} } \right) + x_{i} \left( {p^{0} } \right)}}} \right)_{{q_{k} }} , $$

which is numerically more stable and divisions by 0 are avoided.

Lastly, from Eq. (5), we see that ${r}_{i,j}$ contains the connection coefficients between MRA modules: the direct action of $j$ on $i$ normalized by the ratio ${x}_{j}/{x}_{i}$. Similarly, ${P}_{i,i}$ measures the relative effect of ${q}_{i}$ on ${x}_{i}$. We call it ${q}_{i}$ magnitude. The implicit function theorem provides analytical expressions for ${g}_{i}^{\prime}$ in terms of $f$ partial derivatives, but since $f$ is generally unknown we did not use them. To be rigorous, one should ultimately restrict the mathematical model to a neighborhood of $(x\left({p}^{0}\right),{p}^{0})$ included in all ${V}_{i}$’s, ${W}_{i}$’s, and ${U}_{i}$’s.

MRA-inferred networks have been largely applied for their predictive capabilities ^16^. Let us define a multiple perturbation $q$ to be the linear combination of elementary perturbations ${q}_{k}$. For instance, a perturbation on modules $i$ and $j$ with the same individual magnitudes would be represented by a column vector $c$ with 1’s at positions $i$ and $j$ and 0’s elsewhere. From Eq. (9), we compute

11 $$ \left( {\frac{{{\Delta }x}}{x}} \right)_{q} = - r^{ - 1} Pc, $$

with $\left( {\frac{{{\Delta }x}}{x}} \right)_{q}$ the column vector containing the inferred relative changes on each module activity. Denoting ${\Delta }p$ the parameter changes induced by $q$, the individual module activities are given by

12 $$ \begin{aligned} \left( {\frac{{{\Delta }x_{i} }}{{x_{i} }}} \right)_{q} & = - \left( {r^{ - 1} Pc} \right)_{i} = 2\frac{{x_{i} \left( {p^{0} + {\Delta }p} \right) - x_{i} \left( {p^{0} } \right)}}{{x_{i} \left( {p^{0} + {\Delta }p} \right) + x_{i} \left( {p^{0} } \right)}} \\ & \Leftrightarrow x_{i} \left( {p^{0} + {\Delta }p} \right) = \frac{{x_{i} \left( {p^{0} } \right)}}{{1 + \frac{2}{{\left( {r^{ - 1} Pc} \right)_{i} }}}}\left( {\frac{2}{{\left( {r^{ - 1} Pc} \right)_{i} }} - 1} \right). \\ \end{aligned} $$

If elementary perturbations contribute to different amounts to $q$, the vector $c$ contains ${q}_{k}$’s relative weights. In all cases, linearity between the perturbation strength and its impact on $p$ is assumed.

### Confidence interval estimations

CIs of the MRA parameters were estimated using a bootstrap procedure^22^, an approach that was followed by other authors already^23,24^. In our implementation, we made special efforts to account for the experimental design with biological and technical replicates and to address the difficulty of working with a limited number of replicates. This was achieved by employing tools of statistical process control (SPC) theory^25,26^. SPC is a corpus of statistical methods developed after WW2 to control industrial production processes. One main application is the detection of fluctuations that are not caused by the intrinsic variability of a process. SPC provides estimators of the variance that were optimized for small series (randomly selected samples from a production line in the industrial setting) as well as a procedure to identify the samples outside the expected variability (outliers for our application). SPC can be used for normally distributed data, which will be the case here, but also in a nonparametric manner. In addition to SPC, our bootstrap procedure was also influenced by recent results discussing the propagation of noise in the data across MRA calculations^27^. These results showed that CI estimations on MRA parameters are best obtained by considering the noise on global response coefficients. Accordingly, a global response matrix $R$ is computed for each of the 6 replicates of the complete set of qPCR measures, *i.e.* a sample of 6 values is available for each ${R}_{j,k}$. SPC allows for the estimation of its intra-transfection standard deviation ${s}_{j,k}^{\text{intra}}$ considering the replicate structure: three biological replicates (${k}_{t}=3$) with two technical replicates (${n}_{r}=2$) each. According to SPC methodology, the standard deviation estimator is constructed by computing the mean of the 3 sample ranges, *i.e.*, the mean the absolute values of the differences between the two replicates of each transfection in our particular configuration, divided by Hartley’s constant $d_{2} \left( {n_{r} } \right)$. Namely,

$$ s_{j,k}^{{{\text{intra}}}} = \frac{{\mathop \sum \nolimits_{i = 1}^{{k_{t} }} {\text{Ra}}_{j,k}^{i} }}{{k_{t} d_{2} \left( {n_{r} } \right)}}, $$

with ${\text{Ra}}_{j,k}^{i}=\underset{0\le r\le {n}_{r}}{\mathrm{max}}{R}_{j,k}^{i,r}-\underset{0\le r\le {n}_{r}}{\mathrm{min}}{R}_{j,k}^{i,r}$, where $r$ is the technical replicate index and $i$ the transfection index. Note that ${d}_{2}\left(2\right)=1.128$^28^ (${n}_{r}=2$ here). We did not use SPC to exclude samples outside the expected variability for qPCR data, but we did for the even more sparse RNA-seq data; see the specific Results subsection for the details. Lastly, one million $R$ matrices were generated sampling normal distributions, one distribution for each ${R}_{j,k}$ with mean equal to the average of the 6 raw data replicates and specific variance obtained by SPC. Note that, in practice, different ${R}_{j,k}$ displayed very different variances in some cases on the contrary to Thomaseth et al. study^27^. The 95% CIs were obtained from the 2.5th and 97.5th percentiles. Departure from the assumed normal noise in the data was tested by Kolmogorov–Smirnov tests (data not shown), which detected no significant departure. This does not prove normality (no such test exists in statistics), especially a small number of replicates, but it clearly supports that the estimated 95% CIs are reasonable first approximation.

### Cell culture and perturbation experiments

We used MELN cells, an MCF7-derived cell line stably transfected with the estrogen-responsive luciferase reporter gene ERE-βGlob-Luc-SV-Neo^29^. The cell line was authenticated by short tandem repeat profiling and tested for mycoplasma contamination.

MELN cells were cultured in phenol red-free Dulbecco’s modified Eagle medium (Gibco) containing 5% dextran-charcoal treated FCS (Invitrogen) and antibiotics (Gibco). Perturbations at NRIP1 and LCoR were obtained by siRNAs that were transfected using Interferrin (Polyplus). Perturbations at ERα and RARs were induced by their respective natural ligands: the hormones estrogen (17β-estradiol or E2 for short) and all-trans retinoic acid (RA).

MELN cells were obtained in the following conditions: basal (untreated), E2, RA, E2 + RA, siNRIP1, siLCOR, siNRIP1 + siLCOR, E2 + siNRIP1, E2 + siLCOR, E2 + siNRIP1 + siLCOR, RA + siNRIP1, RA + siLCOR, RA + siNRIP1 + siLCOR, E2 + RA + siNRIP1, E2 + RA + siLCOR, and E2 + RA + siNRIP1 + siLCOR. These experiments were realized in triplicates. We waited 24 h after siRNA transfection before treating with ligands. Cells were harvested after 18 h of culture under ligand stimulation. E2-treated cells received 100 nM E2, RA-treated cells 10 uM RA, and untreated cells ethanol. qPCR measures were realized in duplicates for each of the 3 biological replicates. Validations of the response to E2 and siRNA interference are in Suppl. Fig. 1.

### mRNA quantification

RNA was isolated using “Quick-RNA MiniPrep” (Zymo Research) and reverse transcription (RT)-qPCR assays were done using qScript (VWR) according to the manufacturer’s protocol. Transcripts were quantified using SensiFAST SYBR (BioLine) on an LC480 instrument. The nucleotide sequences of the primers used for real-time PCR were:

RIP140-f (5′- AATGTGCACTTGAGCCATGATG -3′),

RIP140-r (5′- TCGGACACTGGTAAGGCAGG -3′),

LCoR-f (5′- GAACCTAGCGAACAAGACGGTG -3′),

LCoR-r (5′- TGGAGAGTGGCTCAGGGAAGT -3′),

Luciferase-f (5′- CTCACTGAGACTACATCAGC -3′),

Luciferase-r (5′- TCCAGATCCACAACCTTCGC -3′),

HOXA5-f (5′- GCGCAAGCTGCACATAAGTC -3′),

HOXA5-r (5′- GAACTCCTTCTCCAGCTCCA -3′),

ERα-f (5′- TGGAGATCTTCGACATGCTG -3′),

ERα-r (5′- TCCAGAGACTTCAGGGTGCT -3′),

RARα-f (5′- GGATATAGCACACCATCCCC -3′),

RARα-r (5′- TTGTAGATGCGGGGTAGAGG -3′),

PGR-f (5′- CGCGCTCTACCCTGCACTC-3′),

PGR-r (5′-TGAATCCGGCCTCAGGTAGTT-3′).

(RT)-qPCR data are available from the R package.

### mRNA sequencing

For two of the triplicates, in each condition, RNA was extracted as above described. Libraries were prepared with Illumina TruSeq kit and submitted to NextSeq500 sequencing (1 × 75 bp/40 M reads). The first 13 and last 7 bps were cut by an in-house Perl script to eliminate compositional bias. Cut reads were submitted to sickle to eliminate remaining low-quality regions. Alignments were performed against the human genome (hg38) with TopHat v2.10^30^ and read counts extracted with HTSeq-Count^31^. The read count matrix was normalized with edgeR^32^ TMM algorithm. Data are available from GEO under GSE143956.

### aiMeRA library implementation

We implemented the MRA method according to the mathematical formulation above as an R library. (RT)-qPCR data of this project were embedded in the R library for convenience and to provide an example. We also included the data used in the MRA original paper^1^ such that users can check that our code gives the same results as those reported in the latter publication.

## Results

### Transcriptional data

Given ERβ and RARβ expression could not be quantified in MELN cells, we opted for networks involving an ERα module with a reported transcriptional activity by the ERE/luciferase construct, *i.e.*, luciferase mRNA abundance measured ERα activity. ERα mRNA abundance would combine both ligand-bound and free receptors, but only the ligand-bound receptors are relevant for the MRA-inferred network. RARα *versus* RARγ was not distinguished; we estimated their combined transcriptional activity by *HOXA5* gene mRNA abundance, and the corresponding MRA module was named RARs (Fig. 1B). NRIP1 and LCoR activity was determined by their gene mRNA abundance. Given that MELN cells are BC cells, we assumed E2-, RA-, or E2 & RA-stimulated conditions to be basal, *i.e.*, perturbations of ERα and RARs were negative (switch to ethanol condition). Perturbations of NRIP1 and LCoR were achieved by siRNAs, *i.e.*, they were also negative.

### The ERα-NRIP1-LCoR network

**Figure 2:**
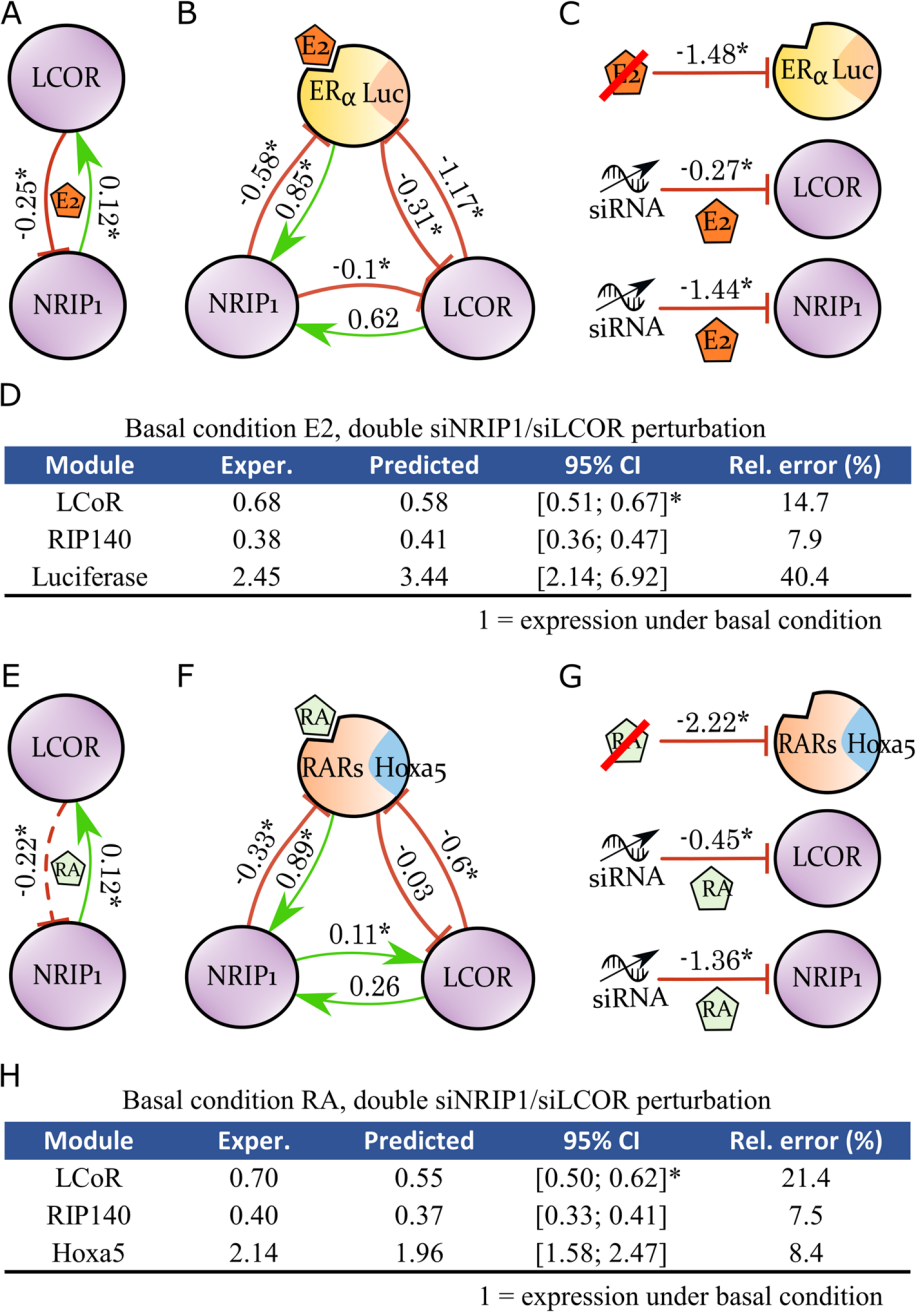
ER and RAR separated networks. (**A**) Connection coefficients between the two corepressors under the E2-stimulated condition. A 95% CI for each MRA parameter was estimated and the parameter denoted by an asterisk provided 0 was not included (sign known with 5% significance). (**B**) The ERα-NRIP1-LCoR transcriptional network. (**C**) ERα, NRIP1, and LCoR perturbation magnitudes. (**D**) Prediction of gene expression under dual siNRIP1 and siLCoR perturbation and E2 stimulation. *Note that LCoR experimental measure lies outside the 95% CI around the predicted value due to higher experimental data variability. A 96% CI equal to [0.45; 0.69] included LCoR experimental value. (**E**) Connection coefficients between the two corepressors under the RA-stimulated condition. (**F**) The RARs-NRIP1-LCoR transcriptional network. (**G**) RARs, NRIP1, and LCoR perturbation magnitudes. (**H**) Prediction of gene expression under dual siNRIP1 and siLCoR perturbation and RA stimulation. *LCoR 99% CI around the predicted value is equal to [0.50; 0.70], it includes LCoR experimental value. Figure created with Inkscape 0.92 (www.inkscape.org) and R 3.6 (r-project.org).

In an unstimulated condition (E2 absence), it is well-known that NRIP1 expression induces LCoR expression^9^. We confirmed the presence of this crosstalk under the basal E2-stimulated condition (Fig. 2A); in addition, we also observed a negative connection coefficient of LCoR to NRIP1. This is in accordance with the former assignment of NRIP1 as a direct target of E2-bound ERα, and LCoR one of its corepressors. The global response coefficients in matrix $R$ were obtained comparing NRIP1 and LCoR expression under E2 stimulation with the successive E2 + siLCoR and E2 + siNRIP1 perturbations. Perturbations on ERα were obtained comparing the E2 stimulated condition with the ethanol condition. Next, we tested the ERα-NRIP1-LCoR network under the same E2-stimulated condition (Fig. 2B). We recapitulated the known induction of NRIP1 by ERα with a negative feedback^5^; we also reconstituted the known inhibition of ERα by LCoR^8^. Interestingly, the induction of LCoR upon NRIP1 expression observed in Fig. 2A became an inhibition. Indeed, this apparent contradiction is correct: in the inferred network including ERα (Fig. 2B), a strong double inhibition via ERα acts positively on LCoR and dominates the, now weak, direct negative connection coefficient. In other words, the 2-module NRIP1-LCoR only reflects the global responses, confounded by the missing ERα, whereas the 3-module ERα-NRIP1-LCoR network disentangles the global to local responses. Moreover, this makes sense biologically since there is no direct transcriptional control of LCoR by NRIP1; NRIP1 can only modulate ERα activity. The same reasoning applies to LCoR inhibition of NRIP1 that became an activation, as shown in Fig. 2A. The strong inhibition of ERα by LCoR, which in turn activates NRIP1 in Fig. 2B, results in a strong indirect inhibitory action counterbalancing the moderate direct positive connection coefficient. Perturbation magnitudes are reported in Fig. 2C. Finally, we assessed the validity of the inferred network by checking its predictive power in a validation experiment where the activity of each module was measured under double siNRIP1 & siLCoR perturbation. In Fig. 2D, reasonable fidelity of the predictions was noted, with relative errors proportional to the CI sizes, *i.e.*, with data variability. Connection coefficients whose 95% CI excluded 0 were marked by an asterisk (Fig. 2A,B,E,F); the sign of such coefficients was known at 5% significance. One may consider the removal of non-marked edges because they could be regarded as negligible (although in most cases where 0 lies inside the CI it is rather close to a boundary); such a procedure is outside the scope of this study, but has been investigated by others^16,21^.

### The RARs-NRIP1-LCoR network

Subsequently, we built a RARs-NRIP1-LCoR network under RA stimulation (Fig. 1B). In the absence of RARs in the MRA-inferred network, NRIP1 and LCoR connection coefficients remained similar to that under E2 stimulation (Fig. 2E). This was expected since these two corepressors have the same function. Similar to above, the global response matrix $R$ coefficients comparing gene expression under RA stimulation with the successive RA + siLCoR and RA + siNRIP1 perturbations. In Fig. 2F, including a RARs module in the inferred networks, we reconstituted the induction of NRIP1 expression by RARs, but also the inhibition of RARs expression by NRIP1^10^. The inhibition of RARs by LCoR has already been established^8^. The LCoR-to-NRIP1 connection coefficient changed of sign between Fig. 2E,F. This connection coefficient was found to be weak in both networks, and the change can be explained using the same arguments as for the ERα-NRIP1-LCoR network reported above (stronger negative LCoR-to-RARs and positive RARs-to-NRIP1 connection coefficients counterbalance modest and direct positive connection coefficients to equate the weak and negative direct effect in Fig. 2E). We also recognized that the connection coefficient between LCoR and NRIP1 under RA (Fig. 2F) was similar to that under E2 stimulation (Fig. 2B), except for the weaker NRIP1-to-LCoR connection coefficient. This illustrated again the expected and similar functioning of both shared corepressors. NRIP1 perturbation magnitude remained similar, but a twofold decrease was found upon LCoR perturbation although the same siRNAs were used (Fig. 2G). Predictions supported the accuracy of the MRA-derived network in a second validation experiment (Fig. 2H).

### The entire ERα-RARs-NRIP1-LCoR network

**Figure 3:**
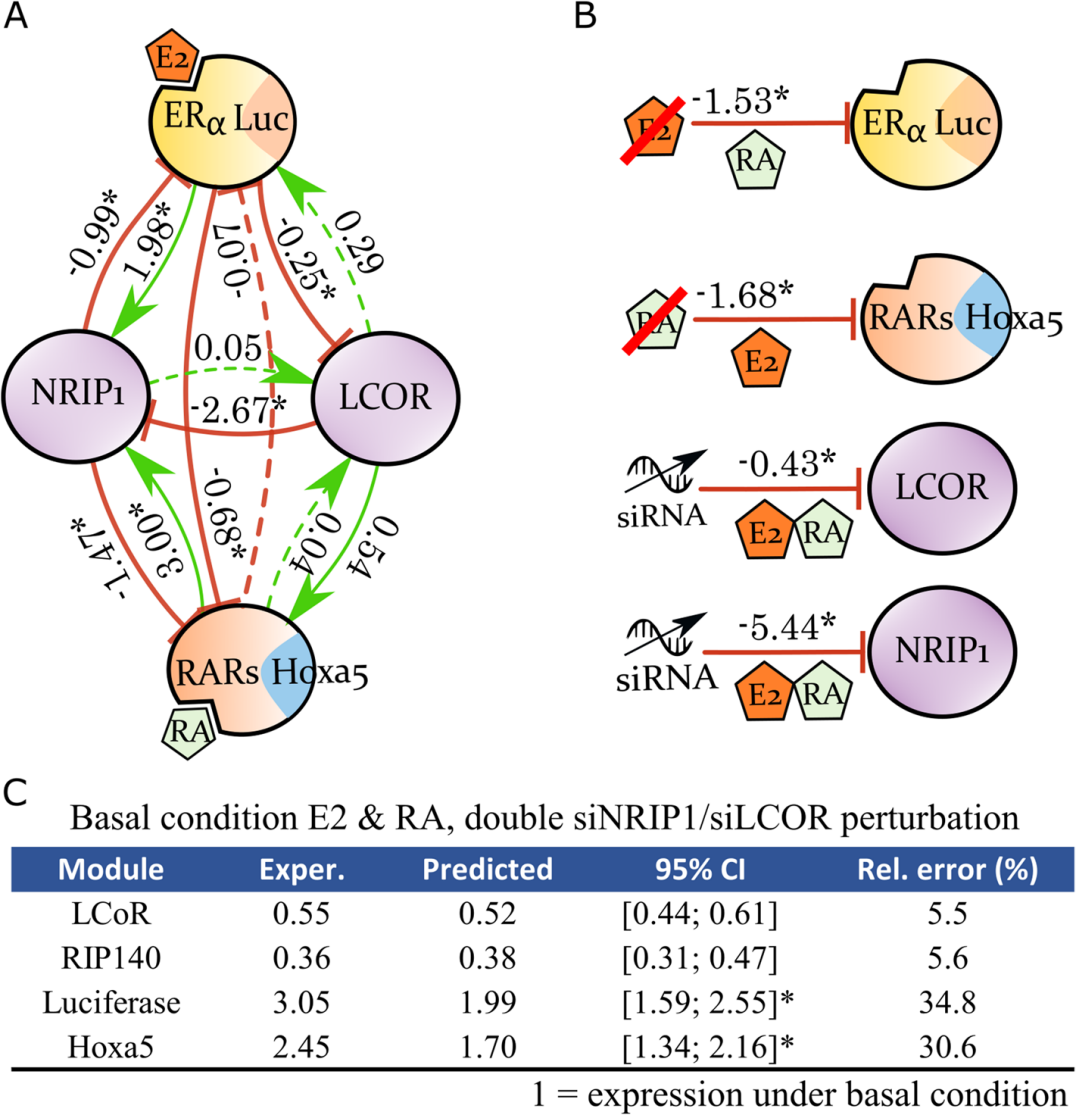
The ERα-RARs-NRIP1-LCoR network. (**A**) MRA-inferred network under dual E2 & RA stimulation. (**B**) Perturbation magnitudes with respect to the basal state E2 & RA stimulation (perturbations on ligands were determined by suppressing one of the two stimulations). (**C**) Predicted activity of the modules upon double siRNA inhibition of NRIP1 and LCoR. *Note that luciferase lies outside its 95% CI due to higher experimental data variability. A 98% CI equal to [1.29; 2.46] included luciferase experimental value. Figure created with Inkscape 0.92 (www.inkscape.org) and R 3.6 (r-project.org).

We applied the same approach to infer a full network of ER-RARs crosstalk under dual E2 & RA stimulation (Fig. 3A). The global response coefficient matrix $R$ was obtained comparing gene expression under dual RA + E2 stimulation with perturbations induced by RA + E2 + siNRIP1, RA + E2 + siLCoR, E2 (suppression of RA), and RA (suppression of E2). Perturbation magnitudes were in the same range as those we found before, but they were greater for NRIP1 (Fig. 3B). To our knowledge, the interaction between NRIP1 and LCoR under this particular E2 & RA condition has never been investigated; only the crosstalk between RARs and ER has been reported, as mentioned in the introduction of this article^14,15^. Hence, we first challenged the inferred network by testing its predictive accuracy (Fig. 3C), which was again satisfactory in a third validation experiment.

Interestingly, the cross-inhibition of ER and RARs signaling acted down two pathways. The MRA-inferred network showed a direct inhibition of ER transcriptional activity by the RARs module, which has already been described in the literature^14,15^. Reciprocal inhibition was suggested but not significant in our data. In agreement with our hypothesis, a parallel cross-talk mechanism was found through the induction of NRIP1 expression, which could subsequently repress both RARs and ERα. MRA inference thus supported the coexistence of both phenomena. LCoR reversed its action on NRIP1 compared to the E2 and RA independent conditions. This reversed role could counterbalance the cross-inhibition of both NRs, but the connection coefficients and the much-attenuated induction of LCoR by NRIP1 suggest that it was not the case here.

### MRA-inferred networks from RNA-seq data

**Figure 4:**
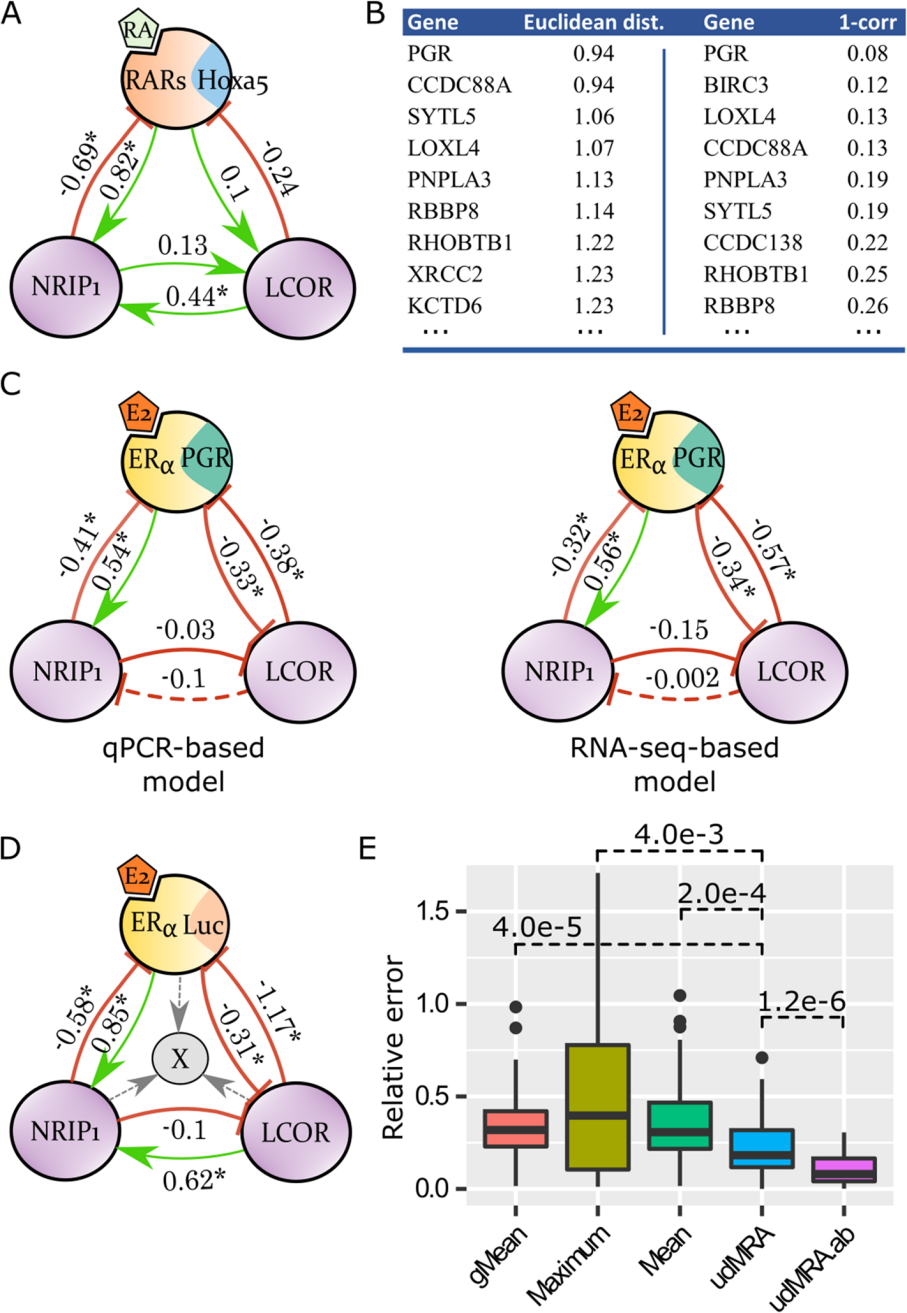
Application to RNA-seq data and genomic predictions. (**A**) RARs-NRIP1-LCoR network inferred from RNA-seq data. (**B**) Nine closest replacement genes for ERE-Luc in the ERα-NRIP1-LCoR network according to the Euclidean distance or 1-correlation. (**C**) ERα-NRIP1-LCoR networks with ERE-Luc replaced by PGR, trained from qPCR and RNA-seq data. (**D**) Principle of unidirectional MRA applications. (**E**) Accuracy of unidirectional MRA predictions (udMRA & udMRA.ab) under the E2 stimulation with double siNRIP1/siLCoR perturbation versus simple predictors (mean, geometric mean (gMean), and maximum of the two siRNAs). Wilcoxon test (n = 60). Figure created with Inkscape 0.92 (www.inkscape.org) and R 3.6 (r-project.org).

Since MRA relies on relative changes of module activity (Eqs. (7, 8)), absolute quantitation is not necessary. Therefore, we inferred a RARs-NRIP1-LCoR network based on the HOXA5*,* NRIP1, and LCoR mRNA abundances that were available in our RNA-seq data (Fig. 4A). Compared with the qPCR-based network shown in Fig. 2F, we observed close values for almost all of the connection coefficients. The only change was the weak LCoR-to-RARs connection coefficient (− 0.03) that became slightly positive (0.13) in the RNA-seq data.

CI estimation based on RNA-seq biological replicates under each condition is challenging. In the bootstrap procedure detailed above, we used a variance estimator adapted to limited numbers of replicates (six qPCR replicates) to conduct point estimations. Given we had only duplicates here, this was no longer an option. Therefore, we applied a common approach used in differential gene analysis to overcome this difficulty, *i.e.*, to learn variance collectively across multiple genes. Practically, our bootstrap method simulates replicates of the $R$ matrix; hence we estimated relative changes of gene expression under the different perturbations investigated (RA-removed, siLCoR, and siNRIP1) *versus* the basal condition (RA stimulation). To have a relevant and sufficient sample of genes for this estimation, we used the 1,092 RA-regulated genes based on edgeR analysis (RA *vs*. ethanol conditions, Suppl. Table 1). Let $G$ be an index set for the 1092 genes. $R$ elements for a given perturbation (one column) are assumed to contain an additive Gaussian noise, which for all practical purposes is essentially correct here. The principle of the algorithm is to use SPC to estimate ${\sigma }_{.,j}$ from all genes under the perturbation ${q}_{j}$, excluding those with replicates that would not be under control according to SPC theory^26,28^. To achieve this, we eliminated such genes iteratively until $G$ no longer changed, as follows:
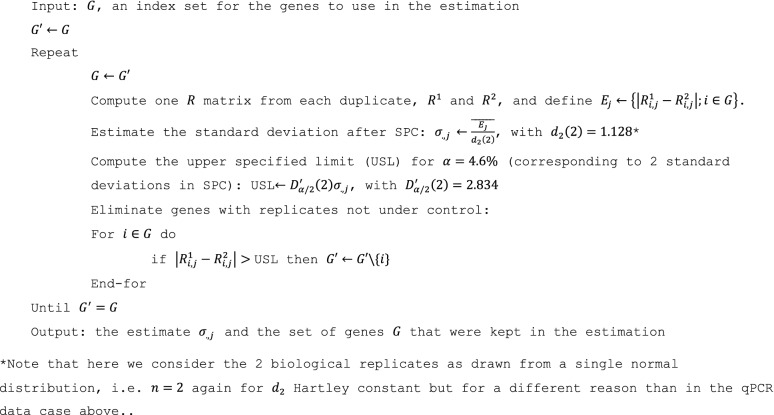


This estimation combined with our bootstrap procedures yielded CIs reported in Suppl. Table 2, and was the basis for marking connection coefficients as significantly different from zero (see Fig. 4A).

### Screening for new genes associated with the ERα-NRIP1-LCoR network

The results above indicated that MRA could be applied to RNA-seq data. We therefore decided to exploit this ability by performing a new type of investigation: we used MRA to identify genes under tight control via the ERα-NRIP1-LCoR network. We reasoned that the MRA-inferred ERα-NRIP1-LCoR network represents a core transcriptional regulator and, in MELN cells, its activity is accurately reported by the ERE-Luc construct. Accordingly, any gene which would behave almost identically to ERE-Luc in terms of expression, would be a natural candidate for tight ERα-NRIP1-LCoR network transcriptional control. We decided to test proximity of genes with ERE-Luc by their ability to yield an MRA-inferred network from RNA-seq data, together with NRIP1 and LCoR, as close as possible to the qPCR data-derived ERα(ERE-Luc)-NRIP1-LCoR network.

We performed intersection of existing ChIP-seq data^33^ with E2-regulated genes from our RNA-seq data (E2 *vs*. ethanol conditions, Suppl. Table 3). This allowed us to identify 884 genes both targeted by ERα and E2-regulated (Suppl. Table 4). Hence, we computed 884 networks with siNRIP1 and siLCoR RNA-seq data, successively replacing ERE-Luc by each of these 884 genes. That is, global response coefficients were obtained comparing NRIP1 and LCoR expression under E2 stimulation with the successive E2 + siLCoR and E2 + siNRIP1 perturbations. Perturbation on the candidate genes were obtained comparing their expression in the presence/absence of E2 stimulation. The genes with the shortest Euclidean distances or 1-correlation between their connection coefficients (the ${r}_{i,j}$ matrix) and those from the original qPCR-based connection coefficients (Fig. 2B) are listed in Fig. 4B. Given the progesterone receptor gene (PGR) ranked first with both measures, we performed a validation experiment by measuring *PGR* expression by qPCR. This enabled us to construct pure qPCR- and pure RNA-seq-based networks, as illustrated in Fig. 4C. These accurately reproduced the original network (Fig. 2B). CI estimations were obtained by applying the method introduced for the RARs-NRIP1-LCoR RNA-seq network; here the variance collected among the 884 E2-regulated/ERα-targeted genes was used.

### Unidirectional MRA on genomic data

We next hypothesized that the ERα-NRIP1-LCoR MRA-derived network could provide means of predicting E2-regulated gene expression. To do this, we introduced a modified, hybrid version of the ERα-NRIP1-LCoR MRA network, including an additional module that cannot influence the other modules (Fig. 4D). The gray unidirectional arrows shown in Fig. 4D represent the connection coefficients between the NRIP1, LCoR, and ERα modules, and the added gene denoted by X. No connection coefficient from X to another module can be estimated due to the absence of perturbation data on X, i.e. we assume that X does not influence NRIP1, LCoR, or ERα transcriptional activity significantly. The connection coefficients from NRIP1, LCoR, or ERα towards X can be learned in the E2-stimulated basal condition by applying elementary perturbations, as described above. One separate network is constructed for each gene X considered. The mRNA abundance of gene X reports an $n + 1$th module activity and, by design, $r_{i,n + 1} = 0$, $\forall i \in \left\{ {1, \ldots ,n} \right\}$, since no perturbation of X is available. From Eq. (7), we can compute $r_{n + 1,j}$, $\forall j \in \left\{ {1, \ldots ,n} \right\}$, by solving the following system:

$$ \left( {\frac{{{\Delta }x_{n + 1} }}{{x_{n + 1} }}} \right)_{{q_{k} }} = \mathop \sum \limits_{j = 1}^{n} r_{n + 1,j} \left( {\frac{{{\Delta }x_{j} }}{{x_{j} }}} \right)_{{q_{k} }} ,\quad k \in \left\{ {1, \ldots ,n} \right\}. $$

The performance of this type of MRA-inferred network that we call udMRA was assessed by its ability to predict the activity of the module $n+1$ under the dual siNRIP1/siLCoR condition, as we did above for the conventional MRA-derived networks (Figs. 2D,H and 3C). To avoid trivially successful predictions on genes that would not vary, we limited the benchmark to the 884 E2-regulated/ERα-targeted genes that were significantly regulated upon siNRIP1 or siLCoR perturbation (under E2 stimulation); 60 genes fulfilled this condition (Suppl. Table 5). For comparison, the relative errors observed after applying udMRA or naïve predictions are depicted in Fig. 4E. udMRA yielded significantly better estimates of X expression, which is consistent with the complex dependency pattern between gene X expression and the modules NRIP1, LCoR, and ERα (Suppl. Fig. 2).

It is worth considering whether perturbation magnitudes during double siRNA interference on the same biological system remain identical; mathematically, this can be formulated as to whether filling the vector $c$ in Eq. (11) with 1 values at the indices of the perturbed module, as conducted so far, is the best option. Equation (11) is written such that we can test different values. We examined the optimal set of coefficients $a$ and $b$ applied to the siNRIP1 and siLCoR perturbations (at the corresponding indices in vector $c$). This was based on the constraint that the prediction errors of luciferase, NRIP1, and LCoR expression (as in Fig. 2D) must be minimal; this yielded $a=1$, and $b=0.4$. Subsequently, these values were used in the udMRA network to more accurately predict the expression of the 60 benchmarked genes. Indeed, the relative error estimates confirmed that this modified inference procedure called udMRA.ab achieved greater accuracy (Fig. 4E).

### aiMeRA usage

**Figure 5:**
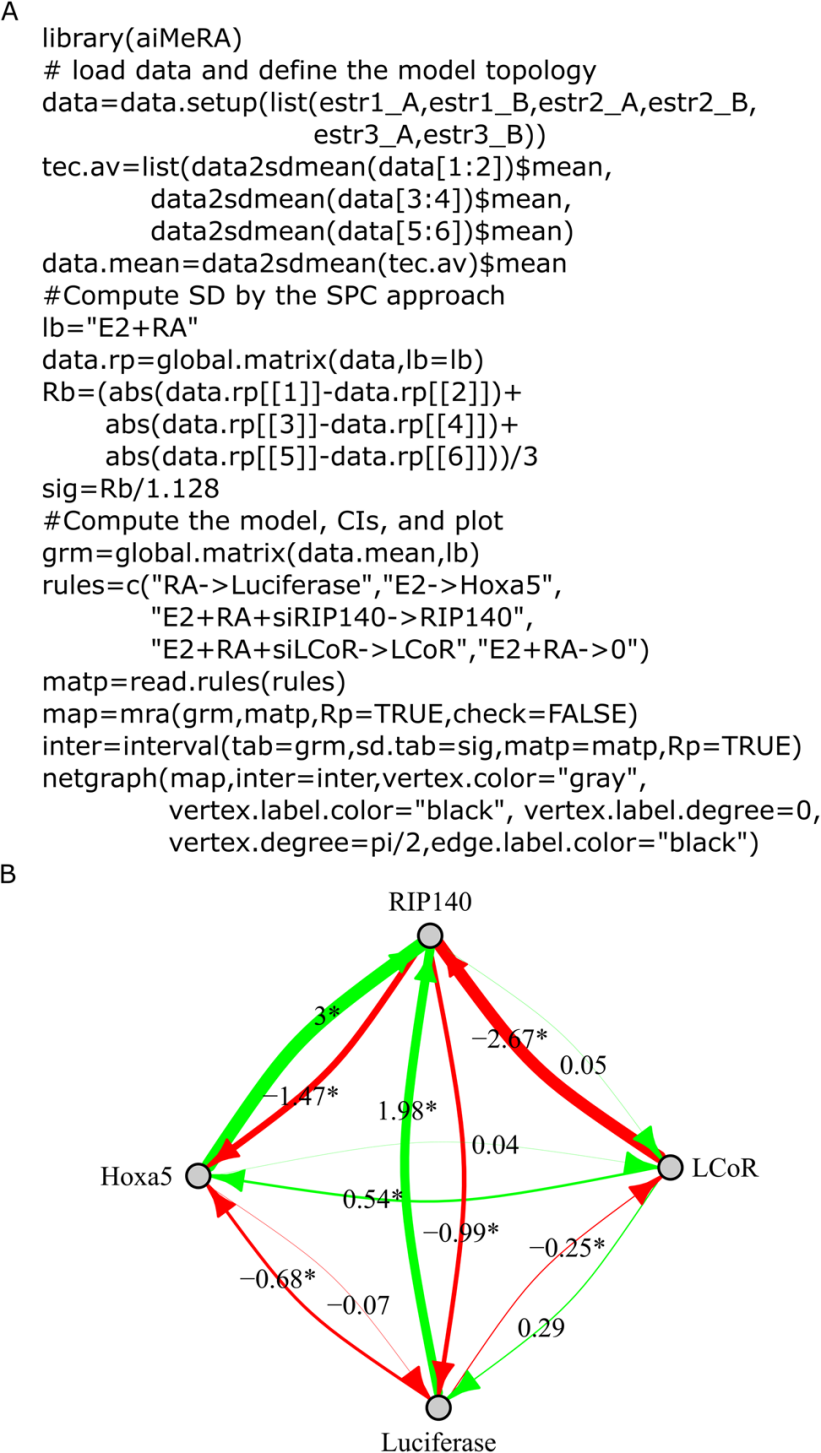
The aiMeRA R package. (**A**) Example R code for loading data, data preparation, and inference of a network. Note that NRIP1 was named by its common alternative name RIP140. Basal condition is E2 & RA stimulation (denoted “E2 + RA- > 0”) and LCoR perturbation is defined as “E2 + RA + siLCoR- > LCoR”. Same logic for RIP140 (= NRIP1). Perturbation on the HOXA5 module reporting RARs activity is defined as E2, *i.e.*, loss of RA stimulation compared to the basal condition was E2 & RA. Perturbation on the luciferase module is similarly defined as RA, *i.e.*, loss of E2. (**B**) Direct plot of an MRA-inferred network in R using the igraph library. Figure created with Inkscape 0.92 (www.inkscape.org) and R 3.6 (r-project.org).

The R package was designed to be generally applicable; it relies on the formulae presented here, and is able to process any quantitative input, including biological and technical replicates. We included a functionality to facilitate the definition of MRA underlying network topologies (Fig. 5A). The design of a network can be specified through the execution of a few generic R functions and network plots can be generated within R directly (Fig. 5B). There is the possibility to export graphs in the graphML or gml formats for loading into Cytoscape^34^ or yED graph editor. More details are provided in the package documentation and Supplementary Material. aiMeRA is available from GitHub and submission to Bioconductor is pending.

## Discussion

MRA inference^1,2^ is a widely used technique to uncover the directions and strengths of connections (connection coefficients or local response coefficients) between components of a biological system—the so-called modules—from systematic perturbation data (Fig. 1). We have developed an R package that implements classical MRA computations along with several extensions devoted to genomic data. The package was entirely implemented in R. The development of our MRA package came along with the opportunity to explore the crosstalk between ER and RAR, two important NRs involved in several tumors, such as BC. We generated unique qPCR and RNA-seq data using BC MELN cells, which are derived from MCF7 cells (see Methods) and a well-established BC model.

The transcriptional activity of ER and RAR is modulated by the shared corepressors NRIP1 and LCoR. Considering networks of growing complexity, *i.e.*, ER-NRIP1-LCoR, RAR-NRIP1-LCoR, and ER-RAR-NRIP1-LCoR, we showed that our MRA inference R package could recapitulate well-known interactions between these molecules by exploiting qPCR data (Figs. 2, 3). We successfully confirmed the known predictive power of MRA-inferred networks as a means to assess their validity in the validation experiments presented. Using original experimental data, the most complex network, ER-RAR-NRIP1-LCoR, enabled us to confront the hypothesis we stated in the Introduction section. Specifically, ER-RAR crosstalk has been described in previous studies as having potential mutual repressive consequences^11–15^. A spatial competition mechanism to bind DNA was proposed in one instance^14^. We reasoned that the sharing of corepressors (NRIP1 and LCoR) may contribute to cross-suppression. Indeed, NRIP1 expression is induced by both ER and RAR upon ligand binding, suggesting that NRIP1 induction by one NR could render NRIP1 available to dump the transcriptional activity of the other NR. The ER-RAR-NRIP1-LCoR network represented in Fig. 3A illustrates both the direct anti-estrogenic activity of RAR, which is in line with current literature, and indirect anti-estrogenic activity mediated by NRIP1, thus substantiating our hypothesis. Reciprocally, ER activity displayed a limited direct inhibition of RAR signaling, but a strong indirect repression via NRIP1 through the same mechanism. In addition, the inhibition of LCoR, which itself inhibits NRIP1, may amplify this phenomenon. This further supports our hypothesis. It is worth noting from Fig. 5 how easy the execution is of such computations in the aiMeRA package, even by a non-specialist.

Following this classical application of MRA, we showed that almost identical MRA-derived networks could be trained from qPCR and RNA-seq data obtained in the same conditions. By employing concepts of SPC theory, we introduced a new procedure that estimates data variance over a gene population when only a few experimental replicates are available. Such a method could be applied with proteomics or phospho-proteomics data when restricted to limited replicates, which is often the case.

Access to genomic data related to the ER-RAR crosstalk motivated an extension of MRA. Since ER acts as a transcription factor upon ligand binding, MRA enabled us to infer a core transcriptional regulatory network around it, *i.e.*, the ER-NRIP1-LCoR network. We thus explored the possibility to identify novel genes under tight control by this network by checking one by one their ability to replace the MELN cell ERE-Luc construct. This construct reports direct ER transcriptional activity. We constructed one MRA-inferred network *per* candidate gene and computed its similarity with the ERE-Luc-based reference network (Fig. 4B). The top candidate was the progesterone receptor gene (*PGR*) that is a widely used reporter of estrogen activity in BC in the clinic. A qPCR-based validation experiment confirmed our prediction (Fig. 4C). This observation supports the potential value of this use of MRA inference to explore unknown associations with a biological network of interest. It also suggests that *PGR* could be employed as surrogate direct reporter of ER transcriptional activity in cell lines devoid of specific constructs, such as ERE-Luc.

We further investigated the possibility of inferring the expression levels of 60 genes that were both E2-regulated and targets of ERα in published ChIP-seq data. This required the construction of hybrid networks, including unidirectional connections to add modules that were not perturbed in the training data set (Fig. 4D). We showed that such hybrid ERα-NRIP1-LCoR networks could outperform naïve predictors (Fig. 4E); this finding was not surprising because the dependencies between the 60 genes tested and the modules of the MRA-inferred network were complex (Suppl. Fig. 2). The accuracy of the predictions could be even further extended by introducing weights in MRA inferences. Other biological systems may be amenable to such modified inferences in the absence of strong feedback loops originating from the inferred genes. Weighted inferences also apply to standard MRA (see our mathematical derivation). Lastly, the inclusion in MRA inferences of molecules that are not perturbed directly and independently has been recently discussed in the special case where conservation laws can be invoked^19^.

The aiMeRA package implements all the extensions discussed in this report.

## Supplementary Information

Supplementary Information.

Supplementary Table 1.

Supplementary Table 3.
