# Supplementary material for: An R package for generic modular response analysis and its application to estrogen and retinoic acid receptor crosstalk
Source: Sci Rep. 2021 Mar 31;11:7272. doi: 10.1038/s41598-021-86544-0 (PMC8012374; doi:10.1038/s41598-021-86544-0)
Supplement: Supplementary file 1 — Supplementary Information. [file 41598_2021_86544_MOESM1_ESM.pdf]

Supplementary Material

An R Package for Generic Modular Response Analysis and its Application to Estrogen and Retinoic Acid Receptor Crosstalk

Gabriel Jimenez-Dominguez[1,2,3], Patrice Ravel[1,2,3], Stéphan Jalaguier[1,2,3], Vincent Cavaillès[1,2,3,*], and Jacques Colinge[1,2,3,*]

[1]Inserm U1194, Institut de Recherche en Cancérologie de Montpellier, 34298 Montpellier, France, [2]University of Montpellier, 34070 Montpellier, France, [3]ICM, Institut régional du Cancer de Montpellier, 34298 Montpellier, France

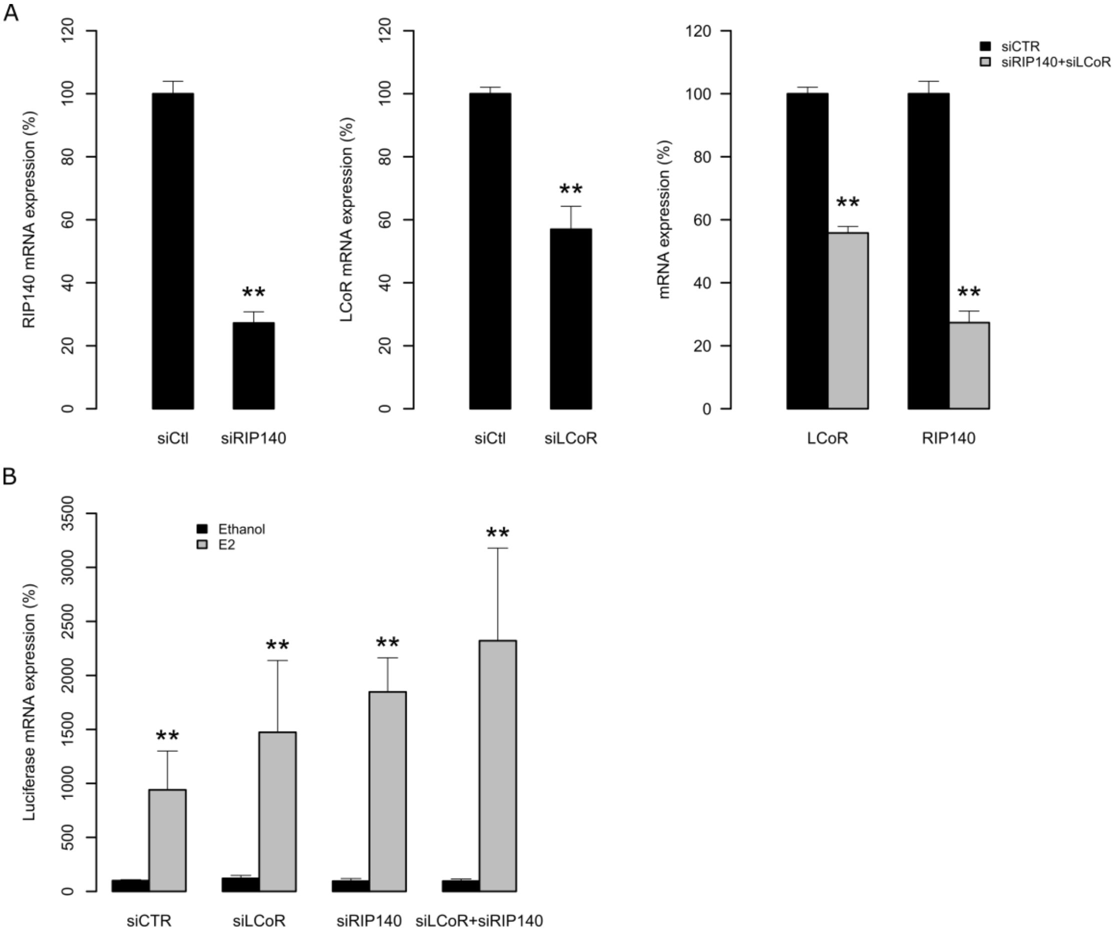

Suppl. Fig. 1. A. MELN cells were transfected with either a siRIP140 (siNRIP1), a siLCoR, or a combination of both siRNAs. RIP140 and LCoR mRNA levels were quantified by real-time qPCR. Results were normalized to 28S mRNA and cells transfected with control siRNA. **B.** MELN cells were transfected as described in A and treated with estradiol (10^{-7} M) when indicated. Luciferase mRNA expression is quantified as in A. Figure created with Inkscape 0.92 (www.inkscape.org) and R 3.6 (r-project.org).

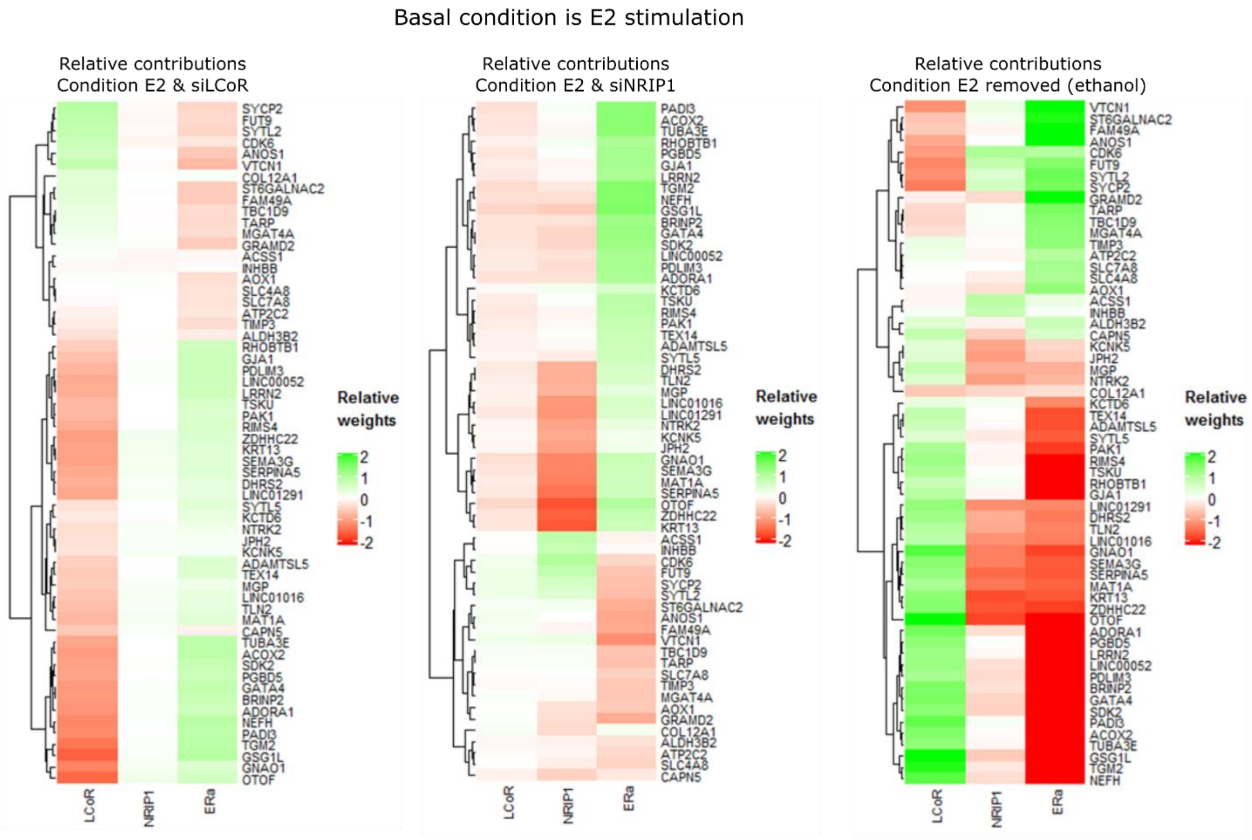

Supplementary Figure 2. Relative contributions of the ERα-NRIP1-LCoR network modules to an added gene X as featured in Fig. 4D. The relative contributions are the product of each module perturbation magnitude and the value of the connection coefficient from this module to gene X, *i.e.*, $r_{i,j}\left(\frac{\Delta x_j}{x_j}\right)_{q_k}$ after our notations. Those relative contributions are represented under each condition involved in the udMRA model training. Marked differences and heterogeneous patterns indicate that the dependency between X expression and the modules is nontrivial. Figure created with Inkscape 0.92 (www.inkscape.org) and R 3.6 (r-project.org).

Supplementary Table 1. The list of 1,092 genes found differentially expressed RA *versus* ethanol by edgeR analysis (P-value<0.01, log_2 fold change > 1 in absolute value, ¾ samples with > 20 read counts) is available as supplementary material file.

Supplementary Table 2. Confidence intervals (95%) for the network in Fig. 4A.

Edge	RNA-seq
LCoR → NRIP1	[0.12;0.19]
LCoR → HOXA5	[-0.58;0.09]
NRIP1 → LCoR	[-0.07;0.32]
NRIP1 → HOXA5	[-1.16;-0.35]
HOXA5 → LCoR	[-0.09;0.28]
HOXA5 → NRIP1	[0.67;0.98]

Supplementary Table 3. The list of 1,666 genes found differentially expressed E2 *versus* ethanol by edgeR analysis (P-value<0.01, log_2 fold change > 1 in absolute value, ¾ samples with > 20 read counts) is available as supplementary material file.

Supplementary Table 4. The 884 genes that were E2-regulated and found in ERα ChIP-seq data.

A4GALT	C19orf38	CTNND2	FGFR1OP	JAKMIP1	MANEAL	OSBPL7	RBBP8NL	SLC34A3	TMEM150A
ABCA4	C1orf112	CTSD	FHL1	JAM2	MAOA	OSTF1	RBKS	SLC35D2	TMEM150C
ABCB4	C1orf116	CXCL12	FHL2	JPH2	MAP2	OTOF	RBL2	SLC38A1	TMEM171
ABCC3	C1orf226	CXXC5	FIBCD1	KAT2B	MAPK4	OVOL2	RCAN1	SLC3A2	TMEM178B
ABCC5	C1QTNF6	CYB5R1	FKBP4	KCNC1	MASTL	P2RX2	RCL1	SLC44A2	TMEM255B
ABCC6	C3	CYCS	FKBP5	KCNC4	MAT1A	P2RY6	RECQL4	SLC4A8	TMEM45B
ABCG1	C3orf52	CYP26B1	FLJ31356	KCNK2	MB	PADI3	RERG	SLC5A8	TMEM97
ABHD17C	C3orf70	DAB2	FLVCR2	KCNK5	MDGA2	PAK1	RET	SLC6A14	TMPRSS3
ABLIM3	C4orf19	DCAF4	FMN1	KCNK6	ME3	PALD1	RFESD	SLC7A5	TMTC3
ACHE	C5AR2	DDIT4	FOXC1	KCNMA1	MEF2D	PALM2	RFTN1	SLC7A8	TNFRSF11A
ACOT7	C8orf46	DEGS1	FOXN1	KCNQ4	MEIOC	PAQR5	RFTN2	SLC9B2	TNS2
ACOX2	C9orf142	DEGS2	FOXO1	KCTD21-AS1	METTL1	PBX1	RGS6	SMIM5	TNS3
ACPP	C9orf152	DEPTOR	FRAS1	KCTD6	MGAT3	PCAT29	RHOBTB1	SMOC2	TOM1
ACSL3	C9orf66	DHRS2	FRK	KIAA0319L	MGAT4A	PCDH1	RHOBTB3	SMOX	TOM1L2
ACSS1	C9orf72	DIO1	FUT8-AS1	KIAA0513	MGP	PCED1B	RHOD	SMPD1	TP53INP1
ADAM11	CA12	DIRC3	FUT9	KIAA1211L	MICAL1	PCLO	RHOV	SMPD3	TP53INP2
ADAMTS9	CA8	DISC1FP1	GAB1	KIAA1549	MICAL2	PDE4B	RIBC2	SMPDL3A	TPBG
ADAMTSL3	CAB39L	DLC1	GALNT10	KIF12	MIR181A1HG	PDGFB	RIMS4	SMTNL2	TPD52L1
ADAMTSL5	CACNA1D	DLEU1	GALNT7	KIF13B	MIR924HG	PDLIM3	RIN2	SNAI1	TPK1
ADCY3	CACNB2	DMRT2	GATA4	KIFC3	MISP	PDSS1	RND3	SNAP23	TPRG1
ADCY6	CACNG6	DNAH17-AS1	GCK	KLF10	MME	PDZK1	RNF103-CHMP3	SNTB1	TRAK1
ADORA1	CALML3-AS1	DNAI1	GDPD3	KLF9	MMP16	PDZRN3	RNF128	SNX24	TRAM2
AHRR	CAMK1D	DNASE1	GFAP	KLHDC7A	MMP25	PERM1	RNF144B	SNX25	TRERF1
ALAD	CAMK2B	DOK7	GFRA1	KMO	MPPED2	PGBD5	RNF152	SORL1	TRIM54
ALDH3B2	CAMKK1	DONSON	GGT1	KRT13	MTHFD1L	PGF	RNF223	SOX3	TRIM9
ALDH4A1	CANT1	DPF3	GHR	KRT7	MTHFR	PGLYRP2	RNF224	SOX9	TRPC6
ALPK3	CAPN5	DRC3	GJA1	KRT86	MUC1	PGM5	RNF43	SPC25	TSC22D1
AMTN	CAPN8	DSCAM	GJB3	LAMC2	MYB	PGR	RORC	SPDEF	TSC22D3
AMZ1	CAPN9	DSCR8	GLA	LDLRAD3	MYBL2	PHLDB2	RPRM	SPIRE2	TSKU
ANK3	CASC15	DTL	GNAO1	LDLRAD4	MYLIP	PHOSPHO2-KLHL23	RSPO1	SPOCD1	TSPAN1
ANKRD13B	CATSPERB	DUSP10	GPAT3	LEF1	MYLK2	PIM1	RTN1	SPRY1	TTC9
ANKRD2	CAV2	DYNLRB2	GPD1L	LHX4	MYO18A	PKIB	RUNDC3B	SPSB4	TTYH1
ANKRD30B	CBLN3	DYNLT3	GPER1	LIF	MYO1B	PLA2G4C	S100A16	SPTBN5	TUBA1A

ANOS1	CCDC103	E2F1	GPR153	LIMA1	MYO6	PLA2R1	S100P	SPTLC3	TUBA3D
AOX1	CCDC125	EBF4	GPR160	LIMD2	MYO7A	PLAC1	S1PR3	SREBF1	TUBA3E
APBB2	CCDC138	ECH1	GPR20	LIMK2	MYRF	PLCD3	SALL4	SRGAP3	TUBA8
APOL3	CCDC170	EEPD1	GPR68	LIN7A	MZT2A	PLCE1	SAMD11	SRMS	UBE2H
AQP3	CCDC183	EFHC2	GPRC5A	LINC00052	NAB2	PLEC	SASH1	SSBP2	UBE2QL1
ARFGEF3	CCDC88A	EFHD1	GRAMD2	LINC00482	NATD1	PLEKHA6	SCD	ST3GAL1	UBE2T
ARHGAP18	CCL17	EFNA1	GRB7	LINC00936	NAV2	PLEKHF1	SCNN1B	ST6GALNAC2	UCA1
ARHGAP32	CCM2L	EFR3B	GREB1	LINC00963	NAV3	PLEKHF2	SCNN1G	ST8SIA4	UGDH
ARHGAP36	CCNB1IP1	ELF5	GRHL3	LINC01016	NBEA	PLLP	SDC1	SULF1	UHRF1
ARHGEF28	CD109	ELFN2	GRIK3	LINC01166	NBEAL2	PLXNA2	SDC2	SUSD3	UNC119
ARHGEF37	CD82	ELOVL2	GSG1L	LINC01291	NBPF1	PMEL	SDCBP2-AS1	SUSD4	UPP2
ARHGEF6	CDK6	ELOVL2-AS1	HAR1B	LINC01376	NBPF13P	PNPLA3	SDK2	SUSD6	USP31
ARID5B	CDKL5	ELOVL5	HAUS1	LINC01488	NBPF25P	PNPLA7	SEC14L2	SV2C	UTRN
ARL3	CDYL2	EMP1	HEBP1	LINC01588	NBR1	POU2F3	SEC14L5	SVOP	VAMP5
ARMCX3	CELSR2	ENC1	HECTD2-AS1	LINC01599	NCAM2	POU6F1	SECTM1	SVOPL	VAT1L
ARNT2	CEMIP	ENOX1	HEG1	LINC-PINT	NCCRP1	PP14571	SEMA3B	SYBU	VGF
ARRB1	CENPH	ENTPD1	HERC3	LIPH	NCS1	PPARA	SEMA3G	SYCP2	VILL
ASAP3	CENPP	EPAS1	HID1	LITAF	NDRG1	PPFIBP2	SEPP1	SYN3	VIPR1
ATP2A3	CEP78	EPB41L4A	HIST2H2BF	LMO1	NDST1	PPL	SERPINA1	SYNDIG1	VPS13D
ATP2C2	CERK	EPB41L5	HIVEP2	LMOD1	NDUFAF2	PPM1E	SERPINA3	SYNPO	VPS9D1-AS1
ATP6V0A4	CERS2	EPHA10	HOMER2	LNX1	NECAB1	PPP1R3C	SERPINA5	SYTL2	VTCN1
ATP6V1C2	CERS4	EPHA4	HPS3	LOC100049716	NEDD4L	PRKAG2-AS1	SERPINB9	SYTL5	VWF
ATP7B	CFAP206	EPN3	HR	LOC100128361	NEFH	PROM2	SFXN4	TACC1	WDFY3-AS2
ATP8A1	CFAP54	ERBB2	HS6ST3	LOC100133985	NEIL2	PRR15	SGCG	TACC2	WDR76
ATP8B2	CGN	ERBB3	HSH2D	LOC100996634	NELL2	PRSS22	SGK1	TACSTD2	WISP2
ATP9A	CGNL1	ERBB4	HSP90AA1	LOC101054525	NEURL1	PRSS23	SGSM3	TAOK3	WNT9A
ATRNL1	CHD6	ERMP1	HSPA12A	LOC101927181	NFATC4	PRSS8	SH2D3C	TARP	XRCC2
AXIN2	CHN1	ESR1	HSPB8	LOC101927391	NHSL1	PSCA	SH3BP4	TBC1D2	XRCC3
B3GALT4	CHPT1	EXOC3L4	HTR7P1	LOC101927746	NIPAL1	PSD4	SH3BP5	TBC1D9	YPEL2
BACE1	CHST15	F5	IBSP	LOC101928105	NIPAL3	PTGES	SH3TC2	TBX6	YPEL5
BAMBI	CHST8	FABP6	IFFO2	LOC101928233	NKAIN1	PTGR1	SHANK2	TECPR1	ZBTB10
BCAM	CLDN4	FAM105A	IFITM10	LOC101929023	NKILA	PTH1R	SHC3	TERT	ZDHHC22
BCAS1	CLDN9	FAM107B	IFRD1	LOC101930370	NOS1AP	PTK2B	SHC4	TESK2	ZDHHC7
BCL2	CLMN	FAM110B	IGDCC3	LOC102723344	NOV	PTK6	SHISA9	TEX14	ZFHX3
BCL2L14	CLMP	FAM13A	IGFBP4	LOC105378047	NOX5	PTPRJ	SIAH2	TFAP2A	ZG16B
BCL3	CLPSL2	FAM161B	IGFBP5	LOC145694	NPNT	PTPRN2	SIDT2	TFAP2A-AS1	ZIC2
BCL9L	CMSS1	FAM198A	IGSF3	LOC283140	NPR3	PTPRQ	SKA1	TFAP2C	ZMIZ1
BEGAIN	CMYA5	FAM212B-AS1	IL17RB	LOC285696	NQO1	PTPRU	SLC11A2	TFAP4	ZNF488
BFSP1	CNKSR3	FAM214A	IL19	LOC647323	NR3C1	PUS10	SLC12A5	TFF1	ZNF703
BFSP2	COL24A1	FAM234B	IL1R1	LOC729224	NR3C2	PVALB	SLC12A7	TFPI	ZNF704
BHLHE40	COL28A1	FAM25A	IL24	LONRF2	NR5A2	RAB11B-AS1	SLC12A8	TGFA	ZNF750
BHLHE40-AS1	COL2A1	FAM3B	IMPA2	LOXL1	NRIP1	RAB26	SLC16A1	TGFBI	
BIK	COL4A3	FAM49A	INHA	LOXL4	NT5DC3	RAB36	SLC16A5	TGM2	
BIRC3	COL9A2	FAM65A	INHBB	LRG1	NT5E	RAB37	SLC16A9	TH	
BLMH	COMT	FAM65C	INPP5J	LRP10	NTN1	RAB3D	SLC17A5	THOC5	
BLNK	CORO2A	FAM69A	IQGAP2	LRP2	NTN4	RAD51	SLC17A9	THUMPD2	
BMF	CPEB3	FAM83A	IRAIN	LRRC10B	NTNG1	RAD54B	SLC19A2	TIAM1	
BMP7	CPNE4	FAM83B	IRF2BPL	LRRC49	NTRK2	RADIL	SLC1A2	TIMP3	
BOP1	CPNE7	FASN	IRF6	LRRC56	NTSR1	RAI14	SLC22A23	TJP2	
BRI3BP	CRAT	FBLN5	ISM1	LRRK2	NUAK1	RAI2	SLC22A5	TK1	
BRINP2	CREB3L1	FBP1	ITGA9	LRRN2	NUMA1	RAMP1	SLC25A19	TLN2	
BTBD11	CRIM1	FBXL6	ITGAE	LTBP1	NXNL2	RAP1GAP	SLC26A1	TM4SF1-AS1	
BTG1	CSGALNACT1	FBXO32	ITGB2	LTBP2	OBSL1	RAPGEF3	SLC27A2	TMC3-AS1	
C12orf60	CSTA	FCMR	ITGB6	LYPD6	OCEL1	RAPGEFL1	SLC29A1	TMC5	
C12orf75	CT62	FFAR2	ITPK1	LYRM4	ORAI3	RARA	SLC2A1	TMCC3	
C14orf159	CTF1	FGD3	ITPR1	MAG	ORC6	RASGRF1	SLC2A10	TMEM105	
C15orf59	CTNNAL1	FGFBP2	JAK2	MAML2	OSBPL10	RBBP8	SLC2A14	TMEM140	

Supplementary Table 5. The 60 genes used to test gene expression inference by udMRA.

ACOX2	GATA4	MGAT4A	SLC7A8
ACSS1	GJA1	MGP	ST6GALNAC2
ADAMTSL5	GNAO1	NEFH	SYCP2
ADORA1	GRAMD2	NTRK2	SYTL2
ALDH3B2	GSG1L	OTOF	SYTL5
ANOS1	INHBB	PADI3	TARP
AOX1	JPH2	PAK1	TBC1D9
ATP2C2	KCNK5	PDLIM3	TEX14
BRINP2	KCTD6	PGBD5	TGM2
CAPN5	KRT13	RHOBTB1	TIMP3
CDK6	LINC00052	RIMS4	TLN2
COL12A1	LINC01016	SDK2	TSKU
DHRS2	LINC01291	SEMA3G	TUBA3E
FAM49A	LRRN2	SERPINA5	VTCN1
FUT9	MAT1A	SLC4A8	ZDHHC22

Code used to generate the MRA models featured in the paper figures

In every case, we average the technical replicates of each biological replicate before averaging over the biological replicates:

```
data=data.setup(list(estr1_A,estr1_B,estr2_A,estr2_B,estr3_A,estr3_B))
tec.av=list(data2sdmean(data[1:2])$mean,data2sdmean(data[3:4])$mean,
            data2sdmean(data[5:6])$mean)
data.mean=data2sdmean(tec.av)$mean
```

The data is transformed into relative activity changes using the global.matrix() function. Then the perturbation rules are set up according to the basal experimental condition of each network.

Fig 2A

```
lb="E2"
grm=global.matrix(data.mean,lb)
rules=c("E2+siRIP140->RIP140","E2+siLCoR->LCoR","E2->0")
```

Fig 2B

```
lb="E2"
grm=global.matrix(data.mean,lb)
rules=c("Et->Luciferase","E2+siRIP140->RIP140","E2+siLCoR->LCoR","E2->0")
```

Fig 2E

```
lb="RA"
grm=global.matrix(data.mean,lb)
rules=c("RA+siRIP140->RIP140","RA+siLCoR->LCoR","RA->0")
```

Fig 2F

```
lb="RA"
grm=global.matrix(data.mean,lb)
rules=c("Et->Hoxa5","RA+siRIP140->RIP140","RA+siLCoR->LCoR","RA->0")
```

Fig 3A

```
lb="E2+RA"
grm=global.matrix(data.mean,lb)
rules=c("RA->Luciferase","E2->Hoxa5","E2+RA+siRIP140->RIP140",
         "E2+RA+siLCoR->LCoR","E2+RA->0")
```

The perturbation matrix is obtained by using the function:

```
matp=read.rules(rules)
```

and the network connectivity is obtained from the global response matrix using the mra() function :

```
map=mra(grm,matp,Rp=TRUE,check=FALSE)
```

To estimate CIs we first calculate the intra transfection standard deviation of relative activity changes by following the SPC approach:

```
data.rp=global.matrix(data,lb=lb)
Rb=(abs(data.rp[[1]]-data.rp[[2]])+abs(data.rp[[3]]-data.rp[[4]])+abs(data.rp[[5]]-
data.rp[[6]]))/3
sig=Rb/1.128
```

Note: The standard deviation for the E2 basal condition can be found as example in the sd.ex table within the aiMeRA package.

Then CIs are computed and plotted on each connectivity coefficient

```
inter=interval(tab=grm,sd.tab=sig,matp=matp,Rp=TRUE)
netgraph(map,inter=inter)
```

Perturbation magnitudes in figure 2C, 2G and 3B were obtained from the local response matrix, which is one of the elements of the list object `map`.

```
map$local_matrix
```

Inferred values in tables of figures 2D, 2H and 3C were obtained by using the function `ab.mra`.

Fig 2D inferred R matrix

```
rules=c("Et->Luciferase","E2+siRIP140->RIP140","E2+siLCoR->LCoR","E2->0")
matp=read.rules(rules)
aux=ab.mra(grm,matp=matp,pred="E2+siLCoR+siRIP140",pert1="E2+siLCoR",
         pert2="E2+siRIP140",ab=FALSE,Rp=TRUE)
```

Fig 2H inferred R matrix

```
rules=c("RA+siRIP140->RIP140","RA+siLCoR->LCoR","Et->Hoxa5","RA->0")
matp=read.rules(rules)
aux=ab.mra(grm,matp=matp,pred="RA+siLCoR+siRIP140",pert1="RA+siLCoR",
         pert2="RA+siRIP140",ab=FALSE,Rp=TRUE)
```

Fig 3C inferred R matrix

```
rules=c("RA->Luciferase","E2->Hoxa5","E2+RA+siRIP140->RIP140",
          "E2+RA+siLCoR->LCoR","E2+RA->0")
matp=read.rules(rules)
aux=ab.mra(grm,matp=matp,pred="E2+RA+siLCoR+siRIP140",
         pert1="E2+RA+siLCoR", pert2="E2+RA+siRIP140",ab=FALSE,Rp=TRUE)
```

To transform the inferred R matrix into inferred data normalized by the baseline:

```
glb=data.mean[rownames(matp),lb]
(-glb*((aux$inferred+2)/(aux$inferred-2)))/glb
```

For figures **4A** and **4C** the RNA-seq data should be downloaded from the GEO platform. In this case, only two technical replicates were generated. Data was hence averaged on two transcriptomes only.

Fig 4A

```
rules=c("RA+siLCoR->LCOR","RA+siRIP140->NRIP1","Et->HOXA5","RA->0")
matp=read.rules(rules)
```

Fig 4C

```
# qPCR based model. First, averaged data of PGR response to perturbations
# and E2 stimulation is added to the table data.mean
data.mean=rbind(data.mean,PGR=0)
data.mean["PGR",c(1,2,6,10)]=c(0.0134506413,0.1657984640,0.1855754764,
                                 0.2552591633)
rules=c("E2+siLCoR->LCoR","E2+siRIP140->RIP140","Et->PGR",
                      "E2->0")
matp=read.rules(rules)
netgraph(mra(data.mean,matp))
```

```
#RNA-seq based model
rules=read.rules(c("E2+siLCoR->LCOR","E2+siRIP140->NRIP1","Et->PGR","E2->0"))
matp=read.rules(rules)
```

Luciferase substitutions to report ERα transcriptional activity in the table of figure **4B** was produced by the construction of a NRIP1-LCOR-x.gene network using the RNA-seq data. The x.gene was successively taken from the list of 884 genes that were both E2-regulated and ERα targets in ChIP-seq data (Suppl. Table 4).

The network in figure **4D** was obtained by defining a module X that has no individual perturbation but for which incoming connectivity can be retrieved by applying udMRA. This is done by using the term 0->X.module in the perturbation rules.

```
rules=c("Et->Luciferase","E2+siRIP140->RIP140","E2+siLCoR->LCoR","E2->0",
        "0->x.module")
matp=read.rules(rules)
```

Then, the inferred gene expression of combining the two siRIP140 and siLCoR in a E2 stimulated condition is obtained by using the function ab.mra. The later was done for figure **4E** udMRA and udMRA.ab barplots

```
data.mean=rbind(data.mean,X.module) #X.module from RNA-seq data
ab.mra(data.mean,matp=matp,pred="E2+siLCoR+siRIP140",pert1="E2+siLCoR",
        pert2="E2+siRIP140",ab=FALSE) #ab=FALSE option for udMRA
ab.mra(data.mean,matp=matp,pred="E2+siLCoR+siRIP140",pert1="E2+siLCoR",
        pert2="E2+siRIP140") #ab=TRUE default option for udMRA.ab
```
